# Coronary Flow Assessment Using Accelerated 4D Flow MRI With Respiratory Motion Correction

**DOI:** 10.3389/fbioe.2021.725833

**Published:** 2021-08-17

**Authors:** Carmen P. S. Blanken, Eric M. Schrauben, Eva S. Peper, Lukas M. Gottwald, Bram F. Coolen, Diederik F. van Wijk, Jan J. Piek, Gustav J. Strijkers, R. Nils Planken, Pim van Ooij, Aart J. Nederveen

**Affiliations:** ^1^ Department of Radiology and Nuclear Medicine, Amsterdam University Medical Centers, Amsterdam, Netherlands; ^2^ Department of Biomedical Engineering and Physics, Amsterdam University Medical Centers, Amsterdam, Netherlands; ^3^ Department of Cardiology, Noordwest Ziekenhuisgroep, Alkmaar, Netherlands; ^4^ Department of Cardiology, Amsterdam University Medical Centers, Amsterdam, Netherlands

**Keywords:** left coronary artery, blood flow quantification, 4D flow MRI, 2D flow MRI, respiratory motion correction

## Abstract

Magnetic resonance imaging (MRI) can potentially be used for non-invasive screening of patients with stable angina pectoris to identify probable obstructive coronary artery disease. MRI-based coronary blood flow quantification has to date only been performed in a 2D fashion, limiting its clinical applicability. In this study, we propose a framework for coronary blood flow quantification using accelerated 4D flow MRI with respiratory motion correction and compressed sensing image reconstruction. We investigate its feasibility and repeatability in healthy subjects at rest. Fourteen healthy subjects received 8 times-accelerated 4D flow MRI covering the left coronary artery (LCA) with an isotropic spatial resolution of 1.0 mm^3^. Respiratory motion correction was performed based on 1) lung-liver navigator signal, 2) real-time monitoring of foot-head motion of the liver and LCA by a separate acquisition, and 3) rigid image registration to correct for anterior-posterior motion. Time-averaged diastolic LCA flow was determined, as well as time-averaged diastolic maximal velocity (V_MAX_) and diastolic peak velocity (V_PEAK_). 2D flow MRI scans of the LCA were acquired for reference. Scan-rescan repeatability and agreement between 4D flow MRI and 2D flow MRI were assessed in terms of concordance correlation coefficient (CCC) and coefficient of variation (CV). The protocol resulted in good visibility of the LCA in 11 out of 14 subjects (six female, five male, aged 28 ± 4 years). The other 3 subjects were excluded from analysis. Time-averaged diastolic LCA flow measured by 4D flow MRI was 1.30 ± 0.39 ml/s and demonstrated good scan-rescan repeatability (CCC/CV = 0.79/20.4%). Time-averaged diastolic V_MAX_ (17.2 ± 3.0 cm/s) and diastolic V_PEAK_ (24.4 ± 6.5 cm/s) demonstrated moderate repeatability (CCC/CV = 0.52/19.0% and 0.68/23.0%, respectively). 4D flow- and 2D flow-based diastolic LCA flow agreed well (CCC/CV = 0.75/20.1%). Agreement between 4D flow MRI and 2D flow MRI was moderate for both diastolic V_MAX_ and V_PEAK_ (CCC/CV = 0.68/20.3% and 0.53/27.0%, respectively). In conclusion, the proposed framework of accelerated 4D flow MRI equipped with respiratory motion correction and compressed sensing image reconstruction enables repeatable diastolic LCA flow quantification that agrees well with 2D flow MRI.

## Introduction

The clinical evaluation of obstructive coronary artery disease (CAD) relies on a combined approach of catheter-based coronary artery angiography (CAG) and physiological testing with for example fractional flow reserve (FFR) or instantaneous wave-free ratio (iFR). Current guidelines recommend non-invasive testing in patients with stable angina pectoris (SAP) to identify probable obstructive CAD before performing invasive CAG (Fihn et al., 2012; Montalescot et al., 2013).

MRI is a non-invasive, non-ionizing imaging technique that can reliably provide prognostic information in patients with CAD using stress-induced perfusion imaging (Motwani et al., 2018). In fact, MRI provides detailed anatomical information (Albrecht et al., 2018; Bustin et al., 2019; Roy et al., 2021) and can also measure coronary flow (Hofman et al., 1996; Davis et al., 1997; Marcus et al., 1999; Johnson et al., 2008; Zhu et al., 2021), potentially enabling assessment of the coronary flow reserve (CFR). The CFR is a measure for the adaptive capacity of the coronary vascular bed to meet the myocardial oxygen demand during increased oxygen consumption of the myocardium. Large-scale studies have shown that a CFR of less than 2.0 is an independent predictor of cardiac mortality and major adverse cardiac events and has greater prognostic value than FFR (Murthy et al., 2011; van de Hoef et al., 2014; Kato et al., 2017). The potential of MRI to concurrently assess coronary anatomy, CFR and myocardial perfusion makes it a potential screening modality for accurate selection and planning of patients with SAP for percutaneous coronary intervention (PCI).

To date, MRI-based coronary flow quantification has only been reported using 2D flow MRI (Hofman et al., 1996; Davis et al., 1997; Marcus et al., 1999; Johnson et al., 2008), which has limitations for clinical use. It requires prior knowledge of the desired measurement location(s) and its accuracy depends highly on correct planning of the imaging slice, i.e. perpendicular to the vessel, distal to the stenosis of interest. In contrast, 4D flow MRI (time-resolved three-dimensional three-directional phase-contrast MRI) provides volumetric coverage (Markl et al., 2012). Therefore, the acquisition is easy to plan, analysis planes can be placed after image acquisition and the flow can be quantified at multiple locations from a single dataset. Yet, coronary flow quantification using 4D flow MRI has never been reported, presumably because the small size of the coronary arteries necessitates the use of a high spatial resolution (∼1 mm^3^), leading to unrealistically long scan times that make it nearly impossible to avoid patient movement causing image deterioration.

High spatial resolution 4D flow MRI at clinically feasible scan times requires sparse sampling. Pseudo-spiral Cartesian undersampling with compressed sensing image reconstruction has previously made intracranial flow quantification possible with good accuracy and repeatability (Gottwald et al., 2020a). Application of this technique to the coronary arteries is promising, provided that we can correct for respiratory motion.

In the current study, we therefore investigate the feasibility and repeatability of accelerated, high spatial resolution 4D flow MRI with respiratory motion correction for flow quantification in the left coronary artery (LCA) of healthy subjects at rest. We hypothesize that respiratory motion correction results in improved visibility of the LCA compared to non-corrected data, and that 4D flow MRI-based measurements of LCA flow agree well with 2D flow MRI-based measurements.

## Materials and Methods

### Image Acquisition

Fourteen healthy subjects (eight female, six male, aged 28 ± 4 years) underwent cardiac MRI at 3T (Ingenia Philips, Best, the Netherlands). The study was approved by the local institutional review board (METC) of Amsterdam UMC and all participants gave written informed consent. A Dixon cardiac angiogram with isotropic spatial resolution of 1.5 mm^3^ was acquired for planning purposes using electrocardiographic (ECG) gating to mid-diastole and respiratory gating using a lung-liver navigator with an end-expiration acceptance window of 7 mm. Next, a 4D flow MRI acquisition was performed with an isotropic spatial resolution of 1.0 mm^3^, covering the LCA in a 30-mm thick transversal slab. This acquisition was directly followed by a 2D flow MRI acquisition planned perpendicular to the LCA, with a spatial resolution of 1.0 × 1.0 mm^2^ and 6.0 mm slice thickness. For the purpose of repeatability testing, the sequence of the aforementioned 4D and 2D acquisitions was performed once more with identical settings.

4D flow MRI was acquired using 8 times-accelerated pseudo-spiral undersampling (Gottwald et al., 2020b; Peper et al., 2020). Three-directional velocity-encoding sensitivity (VENC) was set to 50 cm/s and retrospective ECG-gating enabled cardiac binning into 24 phases. A pencil beam navigator was played out on the lung-liver interface to monitor respiratory motion at a sampling frequency of 2 Hz. To reject outliers caused by deep inspiration, an acceptance window of 20 mm was employed. Breath-held 2D flow MRI was acquired using parallel imaging with a SENSE factor of 2. Through-plane VENC was set to 35 cm/s (lower than for the 4D flow MRI, since the larger slice thickness causes spatial velocity averaging which in our experience conceals local peak velocities observed in the 4D flow MRI acquisition).

Lastly, a real-time coronal balanced steady state free precession (bSSFP) series was run for ∼50 s to monitor foot-head respiratory motion of the LCA with respect to the motion of the liver. This scan was ECG-triggered to mid-diastole and had a spatial resolution of 2.0 × 2.0 mm^2^ and slice thickness of 8.0 mm.

### Respiratory Motion Correction and Image Reconstruction

**FIGURE 1:**
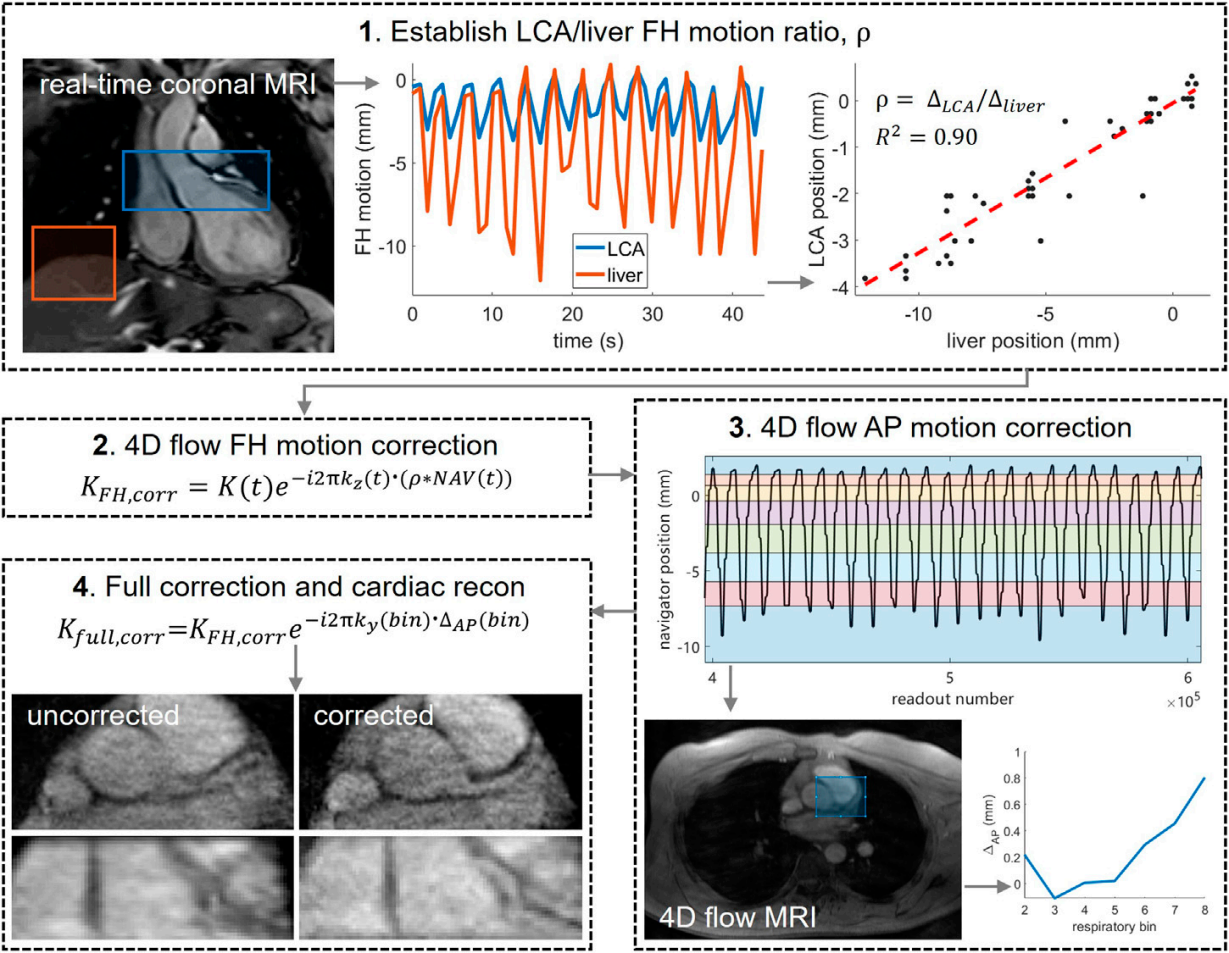
Post-processing pipeline used to correct for respiratory motion in the 4D flow MRI acquisitions. **1)** a real-time ECG-triggered scan was used to determine the ratio ρ between the motion of LCA and liver motion in foot-head (FH) direction. Motion curves were determined by rigid image registration on two separate regions of interest: over the LCA (blue) and over the liver (red). LCA and liver positions were plotted against each other and a linear fit was made, the slope of which is equal to ρ. **2)** LCA offsets, calculated by multiplying liver offsets *NAV*(*t*) with ρ, were converted into time-dependent phase shifts by multiplying the normalized k-space coordinate in the FH direction *k*_*z*_ (t) with the corresponding LCA offset. The complex raw k-space data *K*(t) was then multiplied with these phase shifts. **3)** *NAV*(t) was binned into 8 respiratory phases with equal amounts of data and bin-specific images (time-averaged over mid-diastolic time frames) were reconstructed from the FH motion-corrected raw data. Rigid image registration of bins 2–8 to bin 1 (end-expiration) was performed over a central region including the LCA, producing AP offsets per bin. **4)** AP offsets were corrected in the complex raw k-space data and final image reconstruction was performed.

Prior to reconstruction of the final images, respiration-induced motion of the LCA was corrected in both the foot-head (FH) and anterior-posterior (AP) directions. The methodology is schematically depicted in Figure 1.

#### Motion Correction in Foot-Head Direction

FH motion correction was based on the 4D flow respiratory navigator signal in combination with the real-time coronal scan. In short, the real-time scan was used to determine the ratio ρ between the motion of LCA and liver motion in FH direction, to be able to estimate LCA motion at every k-space readout and correct for it prior to image reconstruction. LCA and liver motion curves were determined by rigid image registration on two separate regions of interest, and their end-expiration heights were aligned. LCA and liver positions were plotted against each other and a linear fit was made, the slope of which is equal to ρ. Next, the lung-liver navigator positions were interpolated to give a position at the time of every imaging readout. The resulting navigator positions will be referred to as *NAV*(t). Offsets from end-expiration were determined and converted into LCA position offsets by multiplication with ρ. To correct for these offsets, readout-specific phase shifts were calculated by multiplying the normalized k-space coordinate in the FH direction *k_z_* (t) with the corresponding LCA offset. The complex raw k-space data *K*(t) was then multiplied with these phase shifts.

#### Motion Correction in Anterior-Posterior Direction

After sorting *NAV*(t) into 8 independent respiratory phase bins with equal amounts of data, bin-specific, time-averaged images were reconstructed from the FH motion-corrected raw 4D flow data, using only k-space samples that were acquired during mid-diastole. From each reconstruction, five central slices were averaged to remove any remaining unresolved FH motion, and rigid image registration of bins 2–8 to bin 1 (end-expiration) was performed over a central region including the LCA. This produced AP offsets per bin, which were corrected in the complex raw k-space data in a similar manner as described for the FH offsets. Right-left offsets were expected to be small and were thus ignored.

#### Image Reconstruction

Compressed sensing image reconstruction was performed in Matlab R2019b (The MathWorks, Inc., Natick, MA), making use of a sparsifying total variation transform in time with a regularization parameter *r* = 0.001 and 20 iteration steps using MRecon (Gyrotools, Zürich, Switzerland) and the Berkeley Advanced Reconstruction Toolbox (BART) (Uecker, 2015). To assess the effect of the respiratory motion correction on the images, non-corrected images were reconstructed as well.

### Data Analysis

**FIGURE 2:**
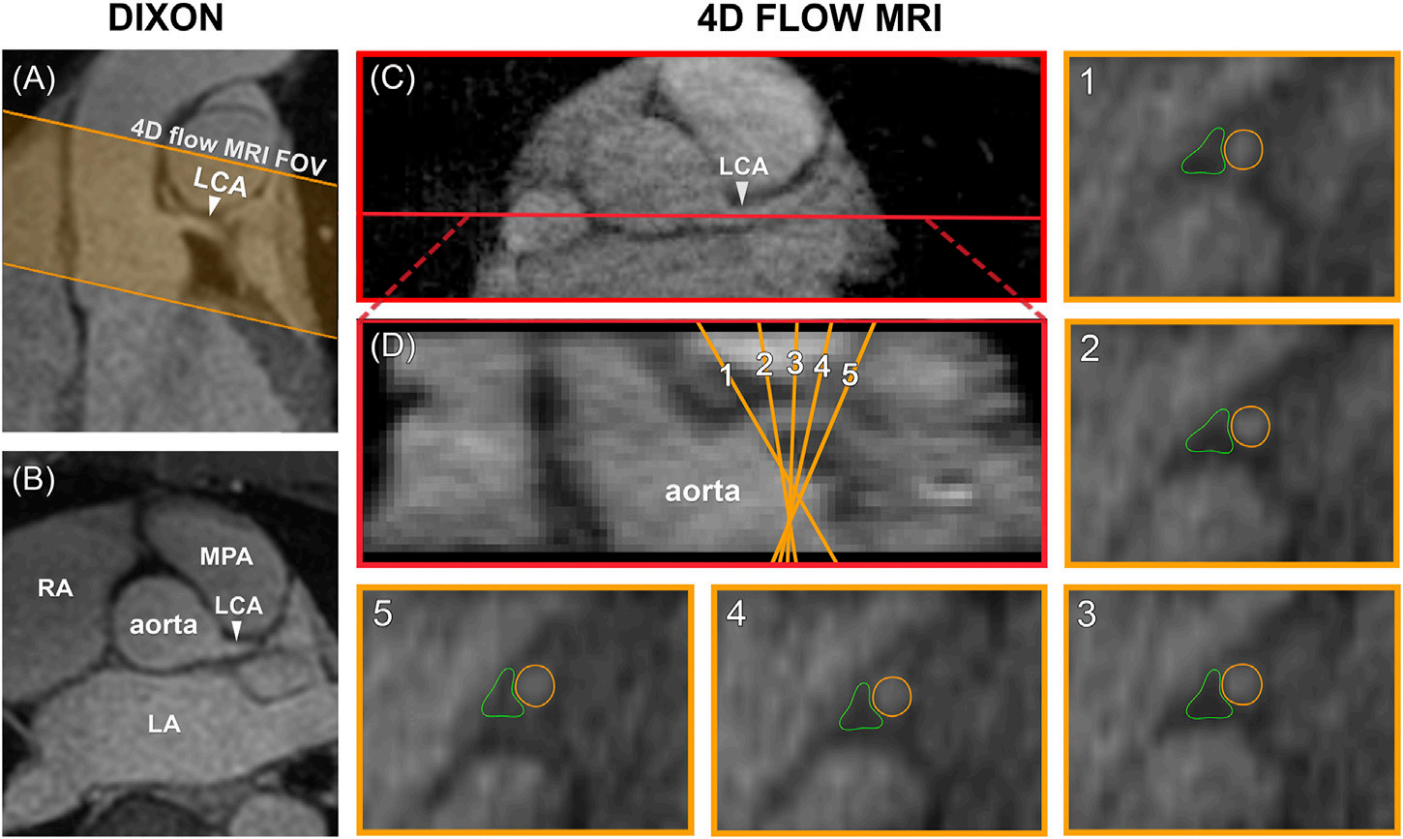
**(A)** Planning of the 4D flow MRI field of view (orange) on the Dixon water image. **(B)** Transversal view of the Dixon water image. **(C)** The LCA is identified on a transversal 4D flow MRI magnitude image and a longitudinal cross-section is made (shown in red). **(D)** The resulting coronal view is used to place five analysis planes perpendicular to the LCA. **1–5)** In these planes, measurement contours are placed around the LCA lumen and in adjacent pericardial fat. FOV = field of view, LCA = left coronary artery, LA = left atrium, RA = right atrium, MPA = main pulmonary artery.

Data analysis was performed in GTFlow V3.2.15 (Gyrotools, Zürich, Switzerland). 4D flow MRI magnitude images were used to localize the LCA branching off from the aorta in a mid-diastolic time frame and to make a longitudinal cross-section, see Figure 2. The longitudinal view was used to place 5 equidistant analysis planes perpendicular to the LCA, approximately 1.5 mm apart, to be able to check for consistency of the measurements over the length of the LCA. Next, the LCA was visually identified in each plane and measurement contours were drawn around the lumen. Additional reference contours were drawn in the adjacent pericardial fat to verify that the measurements would amount to zero flow here, see Figure 2. Both the LCA and the reference contours were copied to all mid-diastolic time frames and onto the corresponding velocity images. Other time frames in which the LCA could not be identified because of blurring due to myocardial contraction and relaxation were discarded. Diastolic flow curves were calculated for each contour, as well as streamlines for visualization. Contour-averaged flow curves were calculated for each subject and averaged over all subjects. For comparison with velocities reported in echocardiographic and 2D flow MRI studies, maximal velocity (V_MAX_) was determined for each contour and each time frame by selecting the voxel with the highest signal within the contour. Next, time-averaged diastolic V_MAX_ and diastolic peak velocity (V_PEAK_) were determined for each subject.

Scan-rescan repeatability of time-averaged diastolic flow, time-averaged diastolic V_MAX_ and diastolic V_PEAK_ was evaluated by means of Bland-Altman analysis, coefficient of variation (CV) and smallest detectable difference. Furthermore, concordance correlation coefficients (CCC) were determined based on absolute agreement and a two-way mixed-effects model (Chen and Barnhart, 2013). CCC was classified as: poor (<0.5), moderate (0.5–0.75), good (0.75–0.9) and excellent (>0.9) (Koo and Li, 2016). CV was defined as the standard deviation of the scan-rescan differences divided by the mean of all scan and rescan measurements. The smallest detectable difference was defined as 1.96 times the standard deviation of the scan-rescan differences. A paired *t*-test was used to compare measured flows and velocities with 2D flow MRI and pericardial fat control measurements. Flow values will be presented as mean ± SD.

## Results

### 4D Flow MRI

**FIGURE 3:**
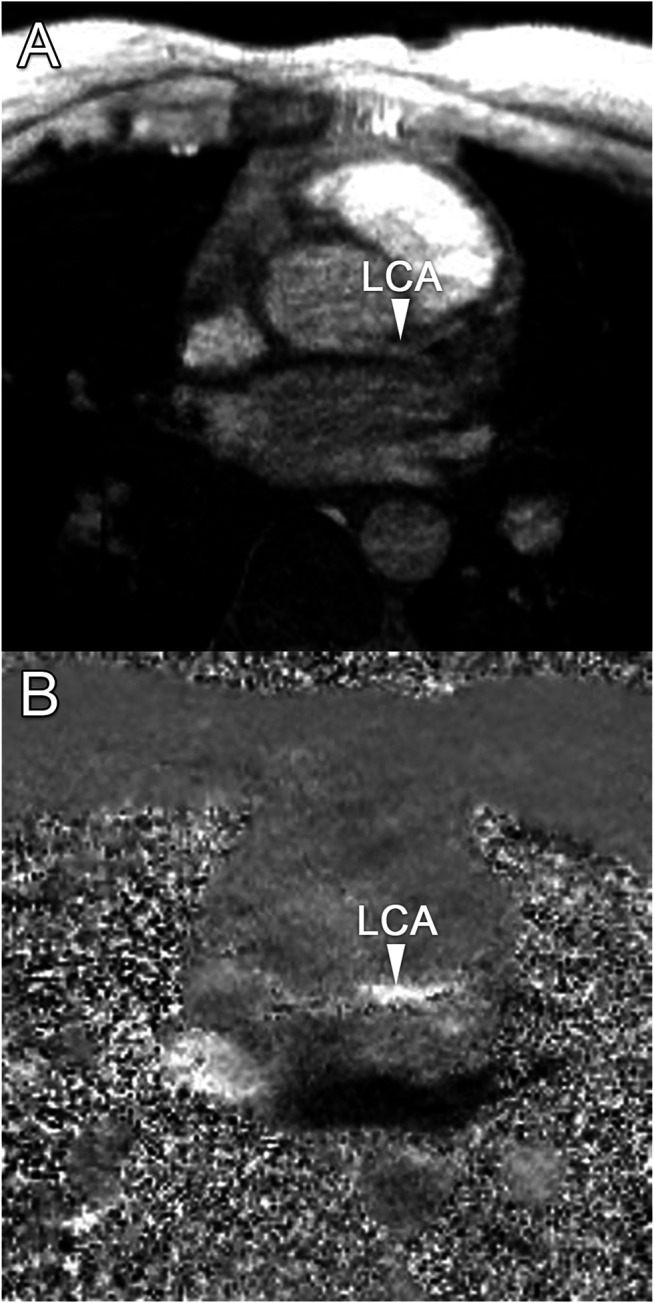
**(A)** Transversal 4D flow MRI magnitude image and **(B)** phase image showing velocities in right-left direction during mid-diastole. Arrows indicate the location of the left coronary artery, where velocity signal can be observed.

**FIGURE 4:**
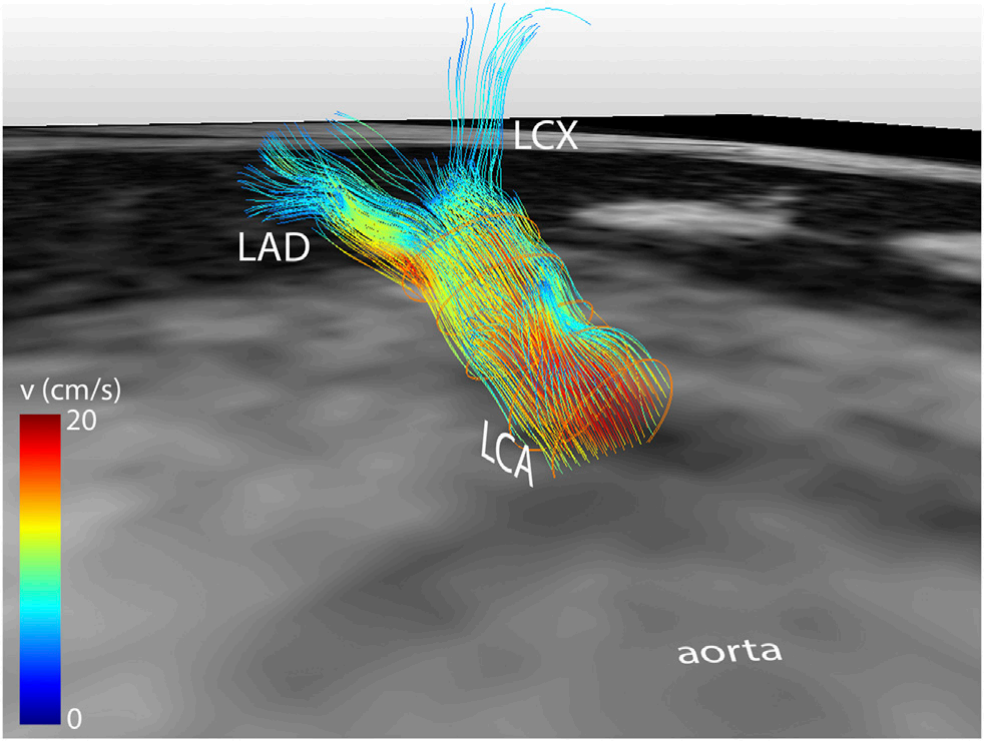
Streamline reconstruction of 4D flow MRI-derived velocities in the LCA for a mid-diastolic time frame. Streamlines initiate from five contours placed in the LCA and split into LAD and LCX. Velocity color-coding shows that the measured velocities in the LAD and LCX are lower than in the LCA.

**FIGURE 5:**
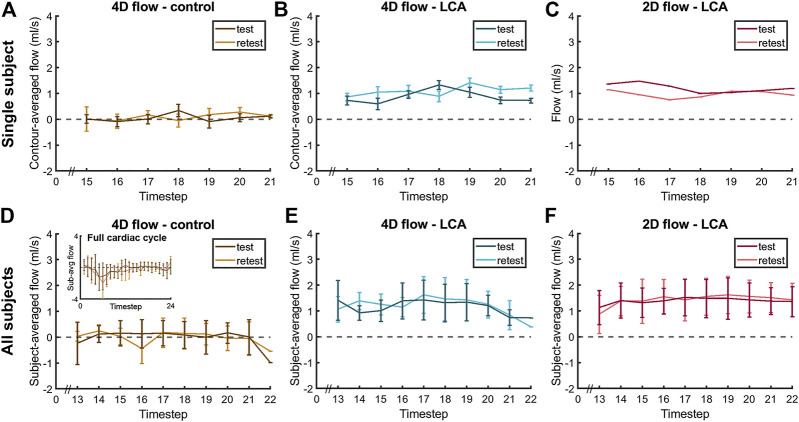
Diastolic flow measured by 4D flow MRI and 2D flow MRI in the LCA and by 4D flow MRI in the adjacent pericardial fat (“4D flow - control”) in a single subject **(A–C)**, same subject as in Figure 4) and averaged over all subjects **(D–F)**, displayed for scan and rescan 4D flow MRI acquisitions. Single-subject flow curves are the result of averaging over all five measurement contours. All-subjects flow curves are the average over all eleven subject-specific (contour-averaged) flow curves.

Median scan time was 12:20 min per 4D flow MRI scan (IQR: 11:30–13:15 min) with a respiratory gating efficiency of approximately 90%. Three subjects were excluded because of insufficient visibility of the LCA in both the original and motion-corrected reconstructions. In these subjects, a pattern of relatively long inspiration phases and no clear skewness towards end-expiration was observed. In the remaining eleven subjects (six female, five male, aged 28 ± 4 years), the LCA was identified in the magnitude images and velocity signal in the phase images. An overview of all original and corrected reconstructions can be found in Supplementary Figure S1. Figure 3 shows example images of phase-contrast magnitude and velocity in right-left direction. Figure 4 shows streamlines in the LCA, splitting into left anterior descending (LAD) and left circumflex (LCX) coronary artery. A video of the streamlines can be found in Supplementary Video S1. Flow curves from this acquisition are presented in Figure 5 (top). Seven out of 24 time frames were examined in this subject. In the other subjects, the number of examined cardiac frames ranged from 5 to 8.

**FIGURE 6:**
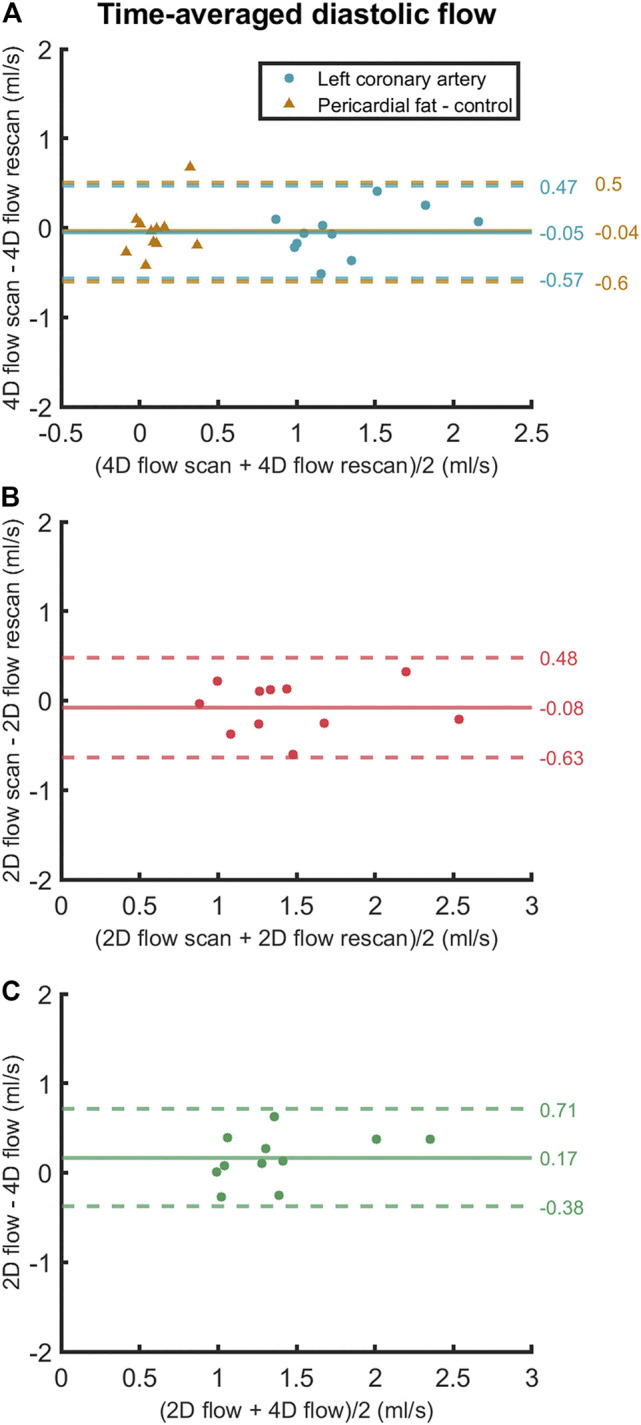
Bland-Altman plots of **(A)** scan and rescan 4D flow MRI measurements, **(B)** scan and rescan 2D flow MRI measurements and **(C)** 4D flow MRI and 2D flow MRI scan-rescan-averaged measurements of time-averaged diastolic LCA flow. Data points are subject-specific. Mean differences and 95% limits of agreement are indicated on the right.

Averaged over all subjects, time-averaged diastolic flows of 1.30 ± 0.39 ml/s in the LCA and 0.11 ± 0.14 ml/s in adjacent pericardial fat were measured, see Figure 5 (bottom). Mean scan-rescan difference and limits of agreement were −0.05 [−0.57; 0.47] ml/s in the LCA - resulting in a smallest detectable difference of 0.52 ml/s - and −0.04 [−0.59; 0.51] ml/s in the pericardial fat (Figure 6). LCA and pericardial fat control measurements of diastolic flow differed significantly (*p* < 0.001). Averaged over all subjects, time-averaged diastolic V_MAX_ in the LCA was 17.2 ± 3.0 cm/s and diastolic V_PEAK_ was 24.4 ± 6.5 cm/s. Statistical results regarding repeatability and agreement between 4D flow MRI and 2D flow MRI are summarized in Table 1. 4D flow-based diastolic LCA flow measurements had good scan-rescan repeatability (CCC = 0.79, CV = 20.4%). Time-averaged diastolic V_MAX_ measurements were moderately repeatable (CCC = 0.52, CV = 19.0%), as were diastolic V_PEAK_ measurements (CCC = 0.68, CV = 23.0%).

**TABLE 1:** Statistical results regarding scan-rescan repeatability and agreement between 4D flow MRI and 2D flow MRI.

	4D flow - LCA		2D flow - LCA		LCA		4D flow - control	
Statistic	Scan	Rescan	Scan	Rescan	4D flow	2D flow	Scan	Rescan
**Time-averaged diastolic flow (ml/s)**								
Mean ± SD [ml/s]	1.27 ± 0.46	1.32 ± 0.36	1.43 ± 0.53	1.51 ± 0.52	1.30 ± 0.39	1.47 ± 0.50	0.09 ± 0.23	0.12 ± 0.15
Mean diff. [ml/s]	−0.05		−0.08		0.17		−0.04	
LOA [ml/s]	−0.57; 0.47		−0.63; 0.48		−0.38; 0.71		−0.59; 0.51	
CV [%]	20.4		19.4		20.1		n/a	
CCC	0.79		0.84		0.75		n/a	
SDD [ml/s]	0.52		0.56		n/a		n/a	
**Time-averaged diastolic V_MAX_ (cm/s)**								
Mean ± SD [cm/s]	17.6 ± 4.0	16.8 ± 2.6	17.3 ± 5.4	18.3 ± 5.9	17.2 ± 3.0	17.8 ± 5.6		
Mean diff. [cm/s]	0.83		−1.03		0.58			
LOA [cm/s]	−5.56; 7.22		−4.92; 2.86		−6.38; 7.54			
CV [%]	19.0		11.2		20.3			
CCC	0.52		0.92		0.68			
SDD [cm/s]	6.39		3.89		n/a			
**Diastolic V_PEAK_ (cm/s)**								
Mean ± SD [cm/s]	24.8 ± 6.7	24.1 ± 7.5	21.9 ± 7.3	22.4 ± 7.4	24.4 ± 6.5	22.1 ± 7.0		
Mean diff. [cm/s]	0.66		−0.50		−2.33			
LOA [cm/s]	−10.37; 11.69		−9.37; 8.38		−14.66; 10.0			
CV [%]	23.0		20.5		27.0			
CCC	0.68		0.81		0.53			
SDD [cm/s]	11.0		8.9		n/a			

LOA, limits of agreement; CV, coefficient of variation; CCC, concordance correlation coefficient; SDD, smallest detectable difference; control, pericardial fat reference measurements.

### 2D Flow MRI

Time-averaged diastolic LCA flow as measured by 2D flow MRI was 1.47 ± 0.50 ml/s (Figure 5). Mean scan-rescan difference and limits of agreement were −0.08 [−0.63; 0.48] ml/s (Figure 6), resulting in a smallest detectable difference of 0.56 ml/s. Time-averaged diastolic V_MAX_ in the LCA was 17.8 ± 5.6 cm/s and diastolic V_PEAK_ was 22.1 ± 7.0 cm/s. 2D flow-based diastolic LCA flow measurements had good scan-rescan repeatability (CCC = 0.84, CV = 19.4%), time-averaged diastolic V_MAX_ measurements were excellently repeatable (CCC = 0.92, CV = 11.2%) and diastolic V_PEAK_ measurements demonstrated good repeatability (CCC = 0.81, CV = 20.5%) (Table 1).

4D flow- and 2D flow-based diastolic LCA flow agreed well (CCC = 0.75, CV = 20.1%) and did not significantly differ (*p* = 0.07), despite a trend towards higher measurements by 2D flow MRI as compared to 4D flow MRI (mean difference and limits of agreement: 0.17 [−0.38; 0.71] ml/s). Moderate agreement and no significant differences between 4D flow MRI and 2D flow MRI were found in measurements of time-averaged diastolic V_MAX_ (CCC = 0.68, CV = 20.3%, *p* = 0.60) and diastolic V_PEAK_ (CCC = 0.53, CV = 27.0%, *p* = 0.25).

## Discussion

In this study, we investigated the feasibility and repeatability of accelerated 4D flow MRI for blood flow quantification in the LCA of healthy subjects at rest. Prospective 8-fold undersampling, respiratory motion correction and compressed sensing image reconstruction facilitated 4D flow MRI-based LCA flow quantification during mid-diastolic time frames. Flow measurements were repeatable and agreed well with 2D flow MRI-based measurements.

CFR assessment is a possible application of the non-invasive LCA flow measurement performed in the current study. Based on the scan-rescan repeatability found in this study, the difference of baseline flow and hyperemic flow should at least be 0.52 ml/s to be detected using the current MRI protocol. Given that the CFR is 4–5 (i.e. an increase from roughly 1.5 to 6.5 ml/s in the LCA) in healthy subjects and around 2 in patients (i.e. an increase from roughly 1.5 to 3.0 ml/s in the LCA), the actual difference will be well above the detection threshold of the presented method.

The observation that 4D flow MRI demonstrates lower repeatability in maximal velocity measurement than 2D flow MRI may be explained by the inherently lower signal-to-noise ratio of the 4D flow MRI acquisition due to smaller voxel size along the length of the LCA (1.0 vs 6.0 mm for 2D flow MRI). Furthermore, unlike 2D flow MRI, 4D flow MRI does not benefit from the slice in-flow effect. The measured velocities were also less repeatable than has been reported for Doppler echocardiography (Hozumi et al., 1998; Kasprzak et al., 2000; Pizzuto et al., 2001; Ciampi et al., 2019).

Literature on healthy LCA diastolic peak velocities is limited. In the early 90’s, studies appeared using transesophageal echocardiography for LCA flow quantification. These studies report baseline values - under general anesthesia - of 29 ± 12 cm/s, 34 ± 8 cm/s and 71 ± 19 cm/s in patients without left main coronary artery stenosis (Yamagishi et al., 1991; Yasu et al., 1993; Kasprzak et al., 2000). More recent studies focus on the LAD using transthoracic echocardiography (Hozumi et al., 1998; Pizzuto et al., 2001; Wittfeldt et al., 2016; Ciampi et al., 2019) or LAD, LCX and RCA using intracoronary Doppler (Ofili et al., 1993; Anderson et al., 2000; van de Hoef et al., 2015) and no longer report LCA velocities. The 24.4 ± 6.5 cm/s (4D flow MRI) and 22.1 ± 7.0 cm/s (2D flow MRI) peak velocities we measured in the LCA are lower than previously reported in studies using transesophageal echocardiography (Yamagishi et al., 1991; Yasu et al., 1993; Kasprzak et al., 2000), but similar to values measured using 2D flow MRI (Schiemann et al., 2006).

Studies using 2D flow MRI that measured LAD - as opposed to LCA - flow have reported time-averaged values of 0.5–1.4 ml/s (Davis et al., 1997; Marcus et al., 1999; Johnson et al., 2008; Zhu et al., 2021). Other studies measured LAD peak flow velocities with 2D flow MRI to determine the CFR and found good correlations with CFR obtained by Doppler guide wire (in patients) and by PET (in healthy subjects) (Hundley et al., 1996; Shibata et al., 1999; Sakuma et al., 2000; Bedaux et al., 2002; Nagel et al., 2003). Interestingly, measured peak velocities were significantly lower by 2D flow MRI than by Doppler guide wire, despite the good correlation between CFRs by the two techniques (Shibata et al., 1999; Bedaux et al., 2002; Nagel et al., 2003; Keegan et al., 2015). These differences were probably a result of the different nature of the two measurements: Doppler guide wire measures velocities along a line whereas in phase-contrast MRI, velocity profiles are spatially smoothed when averaged over the volume of a voxel. Other MRI studies have focused on global CFR assessment based on velocity or flow measurement in the coronary sinus or based on myocardial perfusion by contrast-enhanced MRI (Ibrahim et al., 2002; Lund et al., 2003; Aras et al., 2007; Kato et al., 2017). In short, a variety of studies has reported on coronary flows and velocities, but differences in modalities and anatomical locations of measurement complicate meaningful comparison between studies.

We quantified LCA flow at rest only. For CFR assessment, the flow should also be quantified in the hyperemic state, which may introduce more blurring due to a higher heart rate and heavier breathing, but may also result in higher SNR due to higher velocities and a larger luminal area. Hyperemia can be induced in different ways, the most common being administration of a vasodilatory drug. Another possibility is physical exercise testing with the use of an MRI-compatible ergometer, but this introduces subject motion and has a smaller effect on the myocardial blood flow than a vasodilatory drug. Furthermore, inducing hypercapnia, an increased arterial CO_2_-pressure, with the use of a gas control breathing mask has been shown to have an effect similar to physical exercise (Pelletier-Galarneau et al., 2018).

Not only MRI, but also CT, PET-CT, and myocardial perfusion scintigraphy are potentially available non-invasive techniques to investigate different and sometimes overlapping characteristics of coronary artery disease (CAD) (Heo et al., 2014; Alexanderson-Rosas et al., 2015; Smulders et al., 2017). PET-CT is a powerful technique because it can combine anatomical evaluation and corresponding functional status including coronary flow velocity (reserve) and the assessment of microvascular resistance. However, the limited availability, use of ionizing radiation and costs of PET-CT has prevented its widespread application in clinical practice. MRI may provide a more accessible alternative.

Recent developments in the field of cardiac MRI have enabled whole-heart 5D (4D + a respiratory motion dimension) flow imaging, 5D anatomical imaging of the heart including the coronary arteries, and high-resolution coronary angiography (Feng et al., 2018; Bustin et al., 2019; Ma et al., 2020). The current study is the first to combine and implement high spatial resolution imaging, 3D time-resolved velocity encoding, and 2D respiratory motion correction to achieve coronary flow quantification. Vital to the successful combination of these assets are a number of design elements of the proposed framework. First, the use of pseudo-spiral Cartesian k-space sampling allows for a targeted FH field of view to enable 1.0 mm^3^ resolution at a scan time of approximately 12 min. In contrast, Ma et al. (2020) and Feng et al. (2018) employ a radial phyllotaxis sampling scheme which requires a cubic field of view. This sampling strategy is relatively efficient for high-resolution respiratory motion-resolved whole-heart application (2.5 mm^3^ at a scan time of 8 min, or 1.15 mm^3^ at a scan time of 14 min with the aid of MR contrast), but would require impractically long scan times for 1.0 mm^3^ resolution coronary application. For coronary angiography, Bustin et al. (2019) employed a k-space sampling scheme similar to the current one, however their approach was optimized for diastolic vessel depiction instead of time-resolved flow measurement. Another important design element is the two-dimensional respiratory motion correction based on a one-dimensional navigator in combination with rigid image registration. Multi-dimensional motion correction or resolution is typically achieved using self-navigation (Feng et al., 2018; Bustin et al., 2019; Ma et al., 2020), which requires frequent sampling of the k-space center making it not readily compatible with non-radial sequences. A disadvantage of the 1D navigator-based approach we introduced is the necessary acquisition of an extra scan, prolonging the total scan time by approximately 1 min. Furthermore, our motion correction pipeline is not fully automated and AP motion correction based on image registration requires interim image reconstruction. These aspects further prolong reconstruction time.

The current proof of concept study has a couple of limitations. Firstly, we performed measurements in the LCA only. For meaningful clinical measurements, the approach should be extended to also encompass the LAD and LCX, as well as the right coronary artery (RCA). However, the spatial resolution employed in our study does not allow for accurate measurements in these smaller diameter (2.9–3.9 mm) vessels considering the fact that the luminal area should contain at least 16 voxels to keep the measurement error below 10% (Dodge et al., 1992; Tang et al., 1993). An average LCA has a lumen diameter of 4.5 ± 0.5 mm and fits exactly 16 voxels of the size we used in this study (Dodge et al., 1992). Hence, a higher spatial resolution has to be achieved for clinical application.

Secondly, we only considered diastolic time frames because of the presence of myocardial contraction- and relaxation-induced blurring of the LCA in the systolic images. To resolve this issue, a higher temporal resolution must be achieved while maintaining high spatial resolution. To date, this has only been achieved in single-slice through-plane flow imaging with an efficient k-space sampling scheme (Zhu et al., 2021). Nevertheless, for CFR assessment, diastolic flow values should suffice to determine the ratio between resting flow and hyperemic flow.

Lastly, we tested for repeatability by performing two 4D flow MRI acquisitions in direct succession, without repositioning the subject. Consequently, differences in patient position or acquisition planning were not accounted for.

In general, the main difficulty with high-resolution 4D flow MRI applied to small diameter vessels is that it is prone to motion artifacts, due to the long acquisition time needed. The acquisitions may contain (involuntary) patient movement resulting in image blurring, and breathing motion may induce blurring or ghosting despite respiratory motion compensation, as opposed to single breathhold acquisition used in 2D flow MRI (Dyverfeldt and Ebbers, 2017). A recent advancement, called focused navigation, enables non-rigid image registration in 3D, and can in the future potentially be applied to flow imaging (Roy et al., 2021). Non-Cartesian k-space sampling, in combination with a high temporal resolution, might make the acquisition more robust against motion in general (Glover and Pauly, 1992). This way, systolic time frames might be taken into account as well and flow curves over the entire cardiac cycle can be obtained.

## Conclusion

The proposed framework of accelerated 4D flow MRI with respiratory motion correction and compressed sensing image reconstruction enables non-invasive, diastolic LCA flow quantification that agrees well with 2D flow MRI. Important assets of the developed methodology are the use of pseudo-spiral k-space sampling which allows for a targeted FH field of view, and the 2D respiratory motion correction based on a 1D navigator. Opportunities for further optimization exist in enhancing the temporal resolution, automating the entire reconstruction pipeline, and improving robustness for atypical breathing patterns using more advanced k-space sampling and motion correction schemes. The observed scan-rescan repeatability justifies future experiments on quantification of hyperemic LCA flow, to investigate whether the current acquisition can be used to determine CFR.

## Data Availability

The raw data supporting the conclusions of this article will be made available by the authors, without undue reservation. All 4D flow data were acquired using our in-house developed Amsterdam UMC “PROspective Undersampling in multiple Dimensions” (PROUD) patch. A compiled version of this patch is available on reasonable request.
