# Supplementary figures and images for: Coronary Flow Assessment Using Accelerated 4D Flow MRI With Respiratory Motion Correction
Source: Front Bioeng Biotechnol. 2021 Aug 17;9:725833. doi: 10.3389/fbioe.2021.725833 (PMC8634777; doi:10.3389/fbioe.2021.725833)

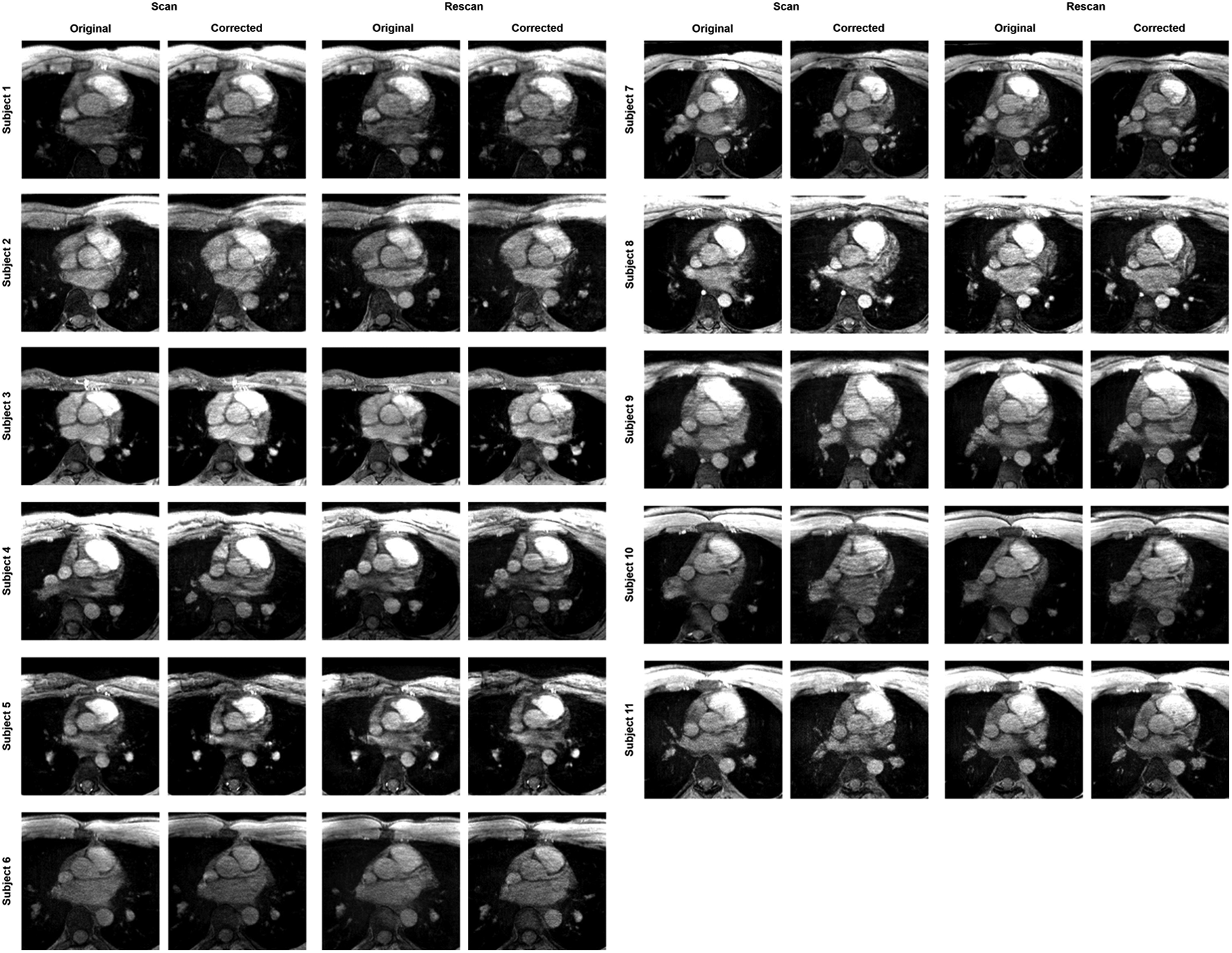

Supplement: Supplementary file 1 [file Image1.tif]
